# Ion Association and Hydration of Some Heavy-Metal Nitrate Salts in Aqueous Solution

**DOI:** 10.1021/acs.jpcb.4c05441

**Published:** 2024-10-03

**Authors:** Johannes Hunger, Richard Buchner, Glenn Hefter

**Affiliations:** † Department for Molecular Spectroscopy, Max Planck Institute for Polymer Research, D-55128 Mainz, Germany; ‡ Institut für Physikalische und Theoretische Chemie, Universität Regensburg, D-93040 Regensburg, Germany; § Chemistry Department, Murdoch University, Murdoch, WA 6150, Australia

## Abstract

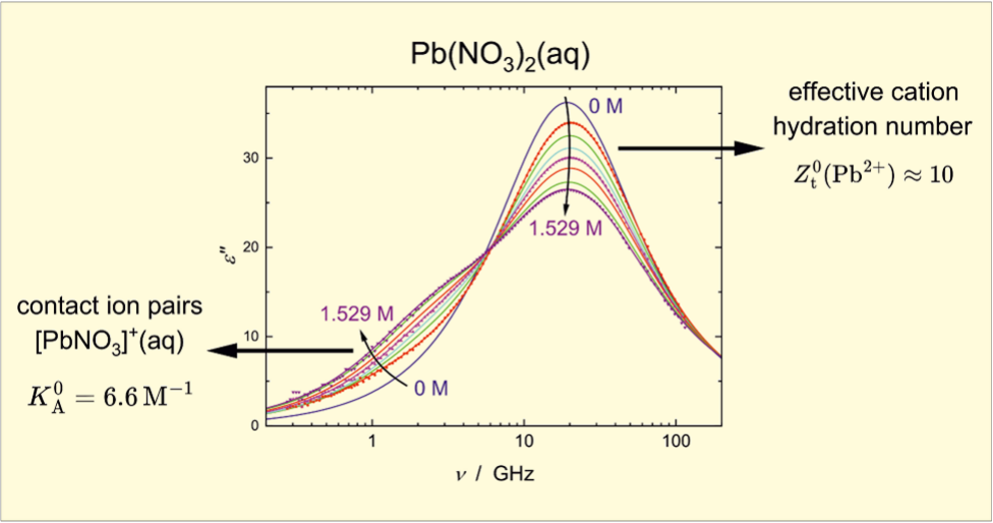

Aqueous solutions of four heavy-metal nitrate salts (AgNO_3_, TlNO_3_, Cd(NO_3_)_2_ and Pb(NO_3_)_2_) have been studied at 25 °C using broadband dielectric relaxation spectroscopy (DRS) at frequencies 0.27 ≤ ν/GHz ≤ 115 over the approximate concentration range 0.2 ≲ *c*/mol L^–1^ ≲ 2.0 (0.08 ≲ *c*/mol L^–1^ ≲ 0.4 for the less-soluble TlNO_3_). The spectra for AgNO_3_, TlNO_3_, and Pb(NO_3_)_2_ were best described by assuming the presence of three relaxation processes. These consisted of one solute-related Debye mode centered at ∼2 GHz and two higher-frequency solvent-related modes: one an intense Cole–Cole mode centered at ∼18 GHz and the other a small-amplitude Debye mode at ∼500 GHz. These modes can be assigned, respectively, to the rotational diffusion of contact ion pairs (CIPs), the cooperative relaxation of solvent water molecules, and its preceding fast H-bond flip. For Cd(NO_3_)_2_ solutions an additional solute-related Debye mode of small-amplitude, centered at ∼0.5 GHz, was required to adequately fit the spectra. This mode was consistent with the presence of small amounts of solvent-shared ion pairs. Detailed analysis of the solvent modes indicated that all the cations are strongly solvated with, at infinite dilution, effective total hydration numbers (*Z*_t_^0^ values) of irrotationally bound water molecules of ∼5 for both Ag^+^ and Tl^+^, ∼10 for Pb^2+^, and ∼20 for Cd^2+^. These results clearly indicate the presence of a partial second hydration shell for Pb^2+^(aq) and an almost complete second shell for Cd^2+^(aq). However, the hydration numbers decline considerably with increasing solute concentration due to ion–ion interactions. Association constants for the formation of contact ion pairs indicated weak complexation that varies in the order: Tl^+^ < Ag^+^ < Pb^2+^ < Cd^2+^, consistent with the charge/radius ratios of the cations and their Gibbs energies of hydration. Where comparisons were possible the present constants mostly agreed well with the rather uncertain literature values.

## Introduction

Heavy metal ions and their salts (“heavy metals,” HMs) have been the focus of much research over many decades, consistent with their technological, scientific, and even (for some) cultural uses.^1−4^ More recently, HMs have attracted interest and concern as ubiquitous and hazardous environmental pollutants spread throughout the planet.^5,6^ Nevertheless, despite their importance, compared with most metal ions, relatively little is known about the behavior of these ions in aqueous solution. Of all the properties of electrolyte solutions perhaps the two most important are the extent of hydration of their constituent ions and the level of interaction (association) between those ions. These two properties, which are to some extent competitive, dominate much of the behavior of all electrolyte solutions.

This paper presents a study of the aqueous solutions of four representative heavy-metal nitrate salts: AgNO_3_, TlNO_3_, Cd(NO_3_)_2_ and Pb(NO_3_)_2_ using dielectric relaxation spectroscopy (DRS). This technique was chosen because it can provide powerful insights into ion hydration, it is sensitive to the existence of weak ion association, and it has an almost unique ability to quantitatively distinguish the three common types of ion pairs (contact, solvent-shared, and double solvent-separated).^7^ Nitrate salts were chosen on the basis of their ready availability in high purity, their good solubilities (with the partial exception of TlNO_3_) and because the nitrate ion, NO_3_^–^, being symmetric does not have a net dipole moment and hence does not contribute to the DR spectra, which simplifies their interpretation. Cations were selected because of indications of unusual coordination characteristics.^8−11^

## Methods

Metal salts AgNO_3_, Carl Roth, p.a. 99.9%; TlNO_3_, Sigma-Aldrich, 99.999% (trace metals basis); Cd(NO_3_)_2_·(H_2_O)_4_, Sigma-Aldrich, 99.997% (trace metals basis); and Pb(NO_3_)_2_, Sigma-Aldrich, 99.999% (trace metals basis) were used as received. Solutions were prepared by weight without buoyancy corrections on an analytical balance, measuring to ±0.1 mg, using high purity deionized water with an electrical resistivity of ≥18 MΩ·cm, drawn from a Millipore line.

Solution densities, ρ, for calculating molar concentrations, *c*/mol L^–1^ (M), were measured at (25 ± 0.05)°C with an accuracy of ca. 0.1 mg·mL^–1^ using a vibrating-tube densimeter (Mettler Toledo DM40) calibrated with N_2_(g) and water, assuming densities from standard sources.^13^ The data obtained for the various solutions are included in Tables S1–S4 of the Supporting Information.

Dielectric spectra of total complex permittivity
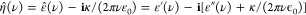
1were measured as a function of field frequency, ν, using frequency domain reflectometers based on flanged open-ended coaxial probes.^14−16^ In eq 1, ε′(ν) is the relative permittivity, ε″(ν) the dielectric loss, and κ the d.c. conductivity of the sample; ϵ_0_ is the electric field constant and **i**^2^ = −1.^17,18^ For all samples, frequencies at 0.83 ≤ ν/GHz ≤ 49 were recorded using coaxial probes based on 1.85 mm feed-throughs. For Cd(NO_3_)_2_(aq) and Pb(NO_3_)_2_(aq) the range 0.27 ≤ ν/GHz ≤ 1.25 was also covered using coaxial probes made from flattened, polished and gold-plated SMA hermetic feed-throughs. Complex scattering parameters were recorded by connecting the coaxial probes to a vector network analyzer (VNA, Anritsu MS4647A). Spectra at 58 ≤ ν/GHz ≤ 115 were recorded using a coaxial probe joined with 1 mm connectors to an external frequency converter (Anritsu 3744A mmW) in combination with the VNA.^19^ Scattering parameters were corrected for systematic errors using air, conductive silver paint, and water as references.^20^ These data were subsequently converted to permittivity spectra using the impedance model described elsewhere.^14,16^ The sample temperature was controlled to (25 ± 1)°C by placing it in a silicon oil bath connected to a circulating thermostat (Julabo F12-ED). Spectra were recorded with 5 mL of the sample in a glass vial in contact with the coaxial probes.

**Figure 1:**
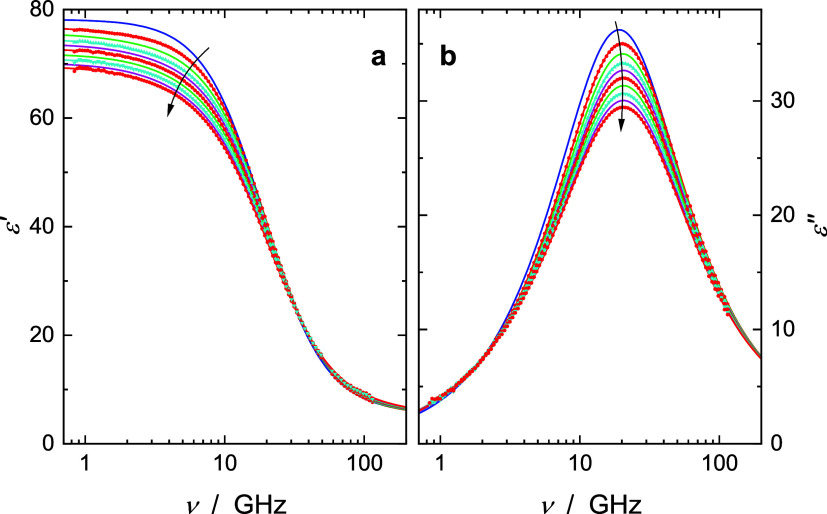
(a) Relative permittivity, ε′(ν), and (b) dielectric loss, ε″(ν), spectra of aqueous solutions of AgNO_3_ at 25 °C (symbols) and their fits with the D+CC+D model (lines). Arrows indicate increasing solute concentrations, *c*/*M* = 0.204, 0.406, 0.604, 0.799, 1.007, 1.242, 1.502, 1.736, 1.994. Experimental points have been partly omitted for visual clarity. For comparison, the spectrum of neat water (*c*/*M* = 0) calculated from the relaxation parameters of Eiberweiser et al.^12^ (*S*_1_ = 72.42, τ_1_ = 8.35 ps, *S*_2_ = 2.43, τ_2_ = 0.278 ps, ε_∞_ = 3.52) is also shown.

For formal description of the spectra so obtained (symbols in Figures 1 and S1–S4), various relaxation models based on the sum of *n* individual relaxation processes
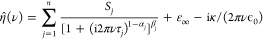
2were tested using a nonlinear least-squares routine that simultaneously fitted ε′(ν) and ε″(ν). In eq 2, ε_∞_ is the high-frequency permittivity of the sample, which is nominally determined only by intramolecular polarizability. Individual dispersion steps, *j* (in order of increasing peak frequency), of amplitude *S*_*j*_, and relaxation time τ_*j*_, were modeled by a Havriliak–Negami (HN) equation with relaxation-time distribution parameters 0 ≤ α_*j*_ < 1 and 0 < β_*j*_ ≤ 1, or its simplified variants: the Cole–Davidson (CD, α_*j*_ = 0), Cole–Cole (CC, β_*j*_ = 1) or Debye (D, α_*j*_ = 0, β_*j*_ = 1) equations.^18^ The static permittivity of the sample is given by ε = ∑ *S*_*j*_ + ε_∞_. Note that in this procedure κ was also treated as an adjustable parameter.

**Figure 2:**
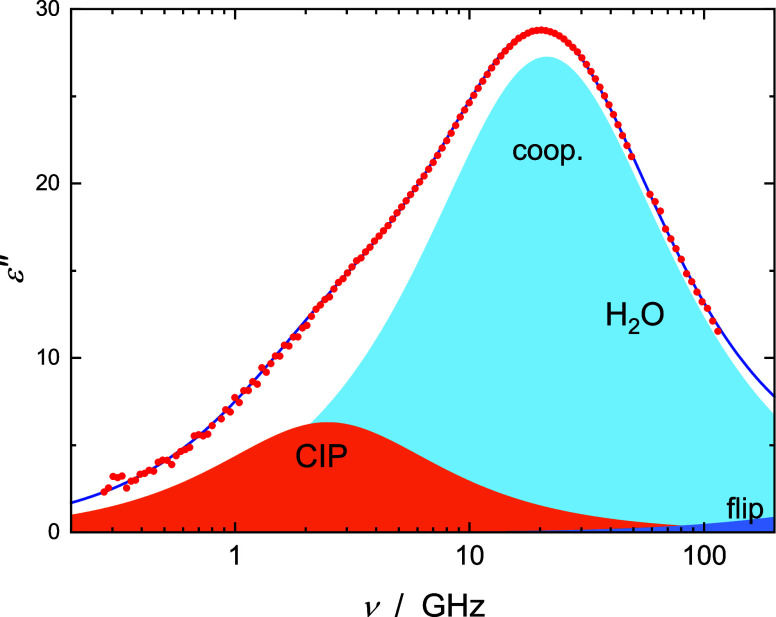
Dielectric loss, ε″(ν), spectrum of a 1.029 M aqueous solution of Pb(NO_3_)_2_ at 25 °C (symbols) and its fit with the D+CC+D model (line). Shaded areas indicate the contributions due to contact ion pairs (CIPs) (*j* = 1) and arising from the cooperative H-bond-network rearrangement process (coop., *j* = 2), and fast H-bond-flip (*j* = 3) of water.

**Figure 3:**
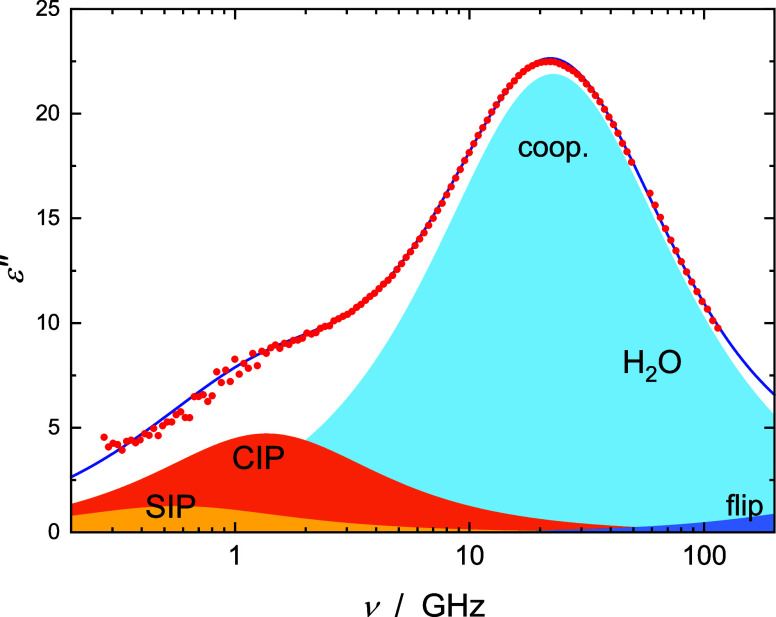
Dielectric loss, ε″(ν), spectrum of 1.293 M aqueous solution of Cd(NO_3_)_2_ at 25 °C (symbols) and its fit with the D+D+CC+D model (line). Shaded areas indicate the contributions of solvent-shared ion pairs (SIPs) (*j* = 1), CIPs (*j* = 2), and water, arising from its cooperative H-bond-network rearrangement process (coop., *j* = 3), and fast H-bond-flip (*j* = 4).

All reasonable models with *n* ≤ 5 were tested and the fits obtained, with their parameters, were scrutinized along the lines described elsewhere.^21^ It was found that for the dielectric spectra of AgNO_3_(aq), TlNO_3_(aq) and Pb(NO_3_)_2_(aq) a D+CC+D model provided the best fit, combining a dominant Cole–Cole mode with ν_2_^peak^ = (2πτ_2_)^−1^ ≈ 18 GHz, and two weak Debye contributions at ν_1_^peak^ ≈ 2 GHz and ν_3_^peak^ ≈ 500 GHz (see Figure 2 as a typical example). For Cd(NO_3_)_2_(aq) a D+D+CC+D model with two weak-to-moderate intensity Debye contributions at ∼0.6 and ∼1.5 GHz provided a superior fit (Figure 3). As discussed below, for all samples the highest-frequency mode can be associated with the fast H-bond flip of solvent water molecules,^22^ although the notable increase in its amplitude for Pb(NO_3_)_2_(aq) (Table S3) and Cd(NO_3_)_2_(aq) (Table S4) may point to additional contributions from ions rattling in their solvent cages.^23^ As this contribution is centered outside the covered frequency range, its relaxation time (0.278 ps) and ε_∞_ (3.52), were fixed in the overall fit to values obtained from the spectrum of neat water extending to 2000 GHz.^12^ The fit parameters obtained are summarized in Tables S1–S4, while the fits are shown as lines in Figures 1 and S1–S4.

Relaxation amplitudes, *S*_*i*_, assigned to a particular dipolar species, *i* (not necessarily identical to a resolved mode, *j*), were evaluated with
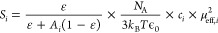
3In this equation *c*_*i*_ is the dipole concentration, μ_eff,*i*_ its effective dipole moment, and *A*_*i*_ the shape-dependent cavity-field factor; *N*_A_, *k*_B_, and ϵ_0_ have their usual meanings.^7,12^

## Results and Discussion

**Figure 4:**
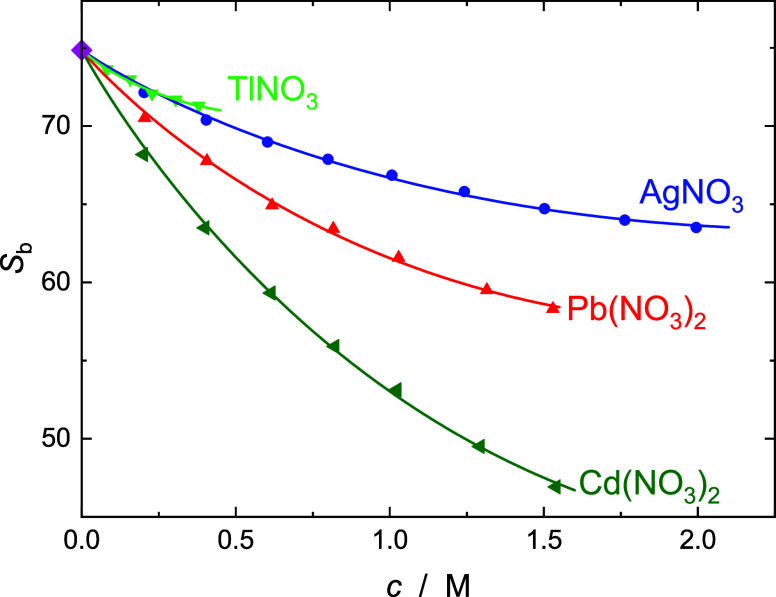
Bulk-water amplitudes, *S*_b_ (symbols), and their fits (lines), of aqueous solutions of AgNO_3_ (blue ○), TlNO_3_ (green ▽), Pb(NO_3_)_2_ (red △), and Cd(NO_3_)_2_ (olive ◁) solutions as a function of solute concentration, *c*, at 25 °C. Value for neat water (diamond) taken from Eiberweiser et al.^12^

Based on the spectrum of neat water,^12,24^ the concentration dependence of the peak frequencies/relaxation times (Figure S5) and their amplitudes (Figure 4), the two highest-frequency modes of all four salts can be straightforwardly assigned to the cooperative rearrangement of the hydrogen-bond network of bulk water (*j* = 2 in the D+CC+D model; *j* = 3 in the D+D+CC+D model) and to its preceding fast H-bond flip (D+CC+D: *j* = 3; D+D+CC+D: *j* = 4).^22^

Based on the concentration dependence of their amplitudes, the lower-frequency modes in the spectra are clearly solute-related. For Cd(NO_3_)_2_(aq) solutions, judging from their τ_1_ values the likely origin of this mode is either ion-cloud relaxation or the reorientation of solvent-shared ion pairs (SIPs). For the other salts, values for τ_1_ (as well as τ_2_ for Cd(NO_3_)_2_)(aq)) suggest contact ion pairs (CIPs) and/or dynamically retarded (slow) hydrating H_2_O molecules.^7^ Evaluation of the relevant amplitudes ruled out both ion-cloud relaxation and slow water as the sources of these lower-frequency modes. Accordingly, these options were not considered further. Ion-related modes contributing to the solution spectra at ν ≳ 100 GHz^23,25−28^ are too weak to be resolved with the present experiments and are thus neglected in the data analysis.

### Ion Hydration

The fast hydrogen-bond flip of individual H_2_O molecules and the subsequent resettlement of the H-bond network of solvent water are connected events.^22^ Neglecting possible weak contributions to *S*_4_ from cage-rattling of the ions,^23^ the relaxation amplitude of (more-or-less) unperturbed bulk water in these solutions is given by *S*_b_ = *S*_2_ + *S*_3_ for the D+CC+D model and by *S*_b_ = *S*_3_ + *S*_4_ for the D+D+CC+D model. As expected from the concentration-dependent electric fields around the ions,^29^ all studied salts exhibit a nonlinear decrease of *S*_b_ with rising concentration. This decrease becomes larger in the sequence TlNO_3_ ≈ AgNO_3_ < Pb(NO_3_)_2_ < Cd(NO_3_)_2_ (Figure 4).

The experimental bulk-water amplitude, *S*_b_(*c*), at salt concentration *c*, can be written as
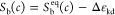
4In eq 4, *S*_b_^eq^(*c*) represents the equilibrium amplitude of the solvent, which is determined by the analytical water concentration, *c*_w_, and by the static (equilibrium) depolarization of the solvent due to the alignment of H_2_O dipoles by the electric field of the ions. From *S*_b_^eq^(*c*) the concentration of DRS-detected bulk water, *c*_b_, is obtained with eq 3.^7^

The second term in eq 4, Δε_kd_, describes the additional kinetic depolarization (kd) of solvent dipoles due to ion migration.^30^ The magnitude of kd is proportional to the solution conductivity, κ, and can be obtained from the equation of Sega et al.^31^
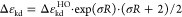
5where
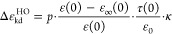
6is the decrement at vanishing salt concentration, given by the corrected continuum model of Hubbard and Onsager (HO),^30^ σ is the reciprocal Debye length at a given ionic strength, *R* is the average effective ion radius, ε(0) and ε_∞_(0) are respectively the static and infinite-frequency permittivities of the neat solvent, τ(0) is the relaxation time of the dominant solvent dispersion step and *p* is a hydrodynamic parameter that accounts for the coupling of translational ion motions to the macroscopic viscosity.

For the present work, slip boundary conditions (*p* = 2/3) and *R* = (*r*_+_ + *r*_–_)/2 were assumed. The required ion radii, *r*_*j*_ (Table S5) were taken from Marcus.^32^Figure S5 illustrates the impact of kinetic depolarization on the resulting effective hydration numbers (see below). The effective dipole moment of 3.746 D, obtained from the data for neat water, was used when calculating *c*_b_(*c*) from *S*_b_^eq^(*c*) with eq 3.^33^

**Figure 5:**
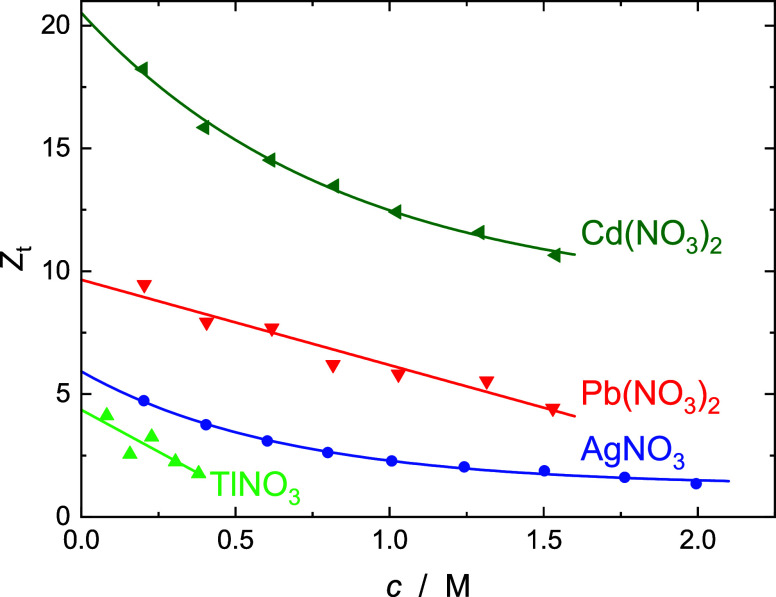
Effective total hydration numbers, *Z*_t_ (symbols), and their fits (lines; see Table 1 for details) for aqueous solutions of AgNO_3_, TlNO_3_, Cd(NO_3_)_2_, and Pb(NO_3_)_2_ at 25 °C.

The thus-obtained effective bulk-water concentrations, *c*_b_, yielded values for the effective total hydration numbers of the salts
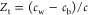
7as the total number of dynamically retarded H_2_O dipoles per equivalent of salt (Figure 5).^7^ Note that *Z*_t_ reflects the amount of apparently missing bulk water but not how strongly it is affected dynamically, i.e., whether the dipoles are “frozen” (irrotationally bound, ib) on the time scale of the experiment, or just slowed down sufficiently to produce a new lower-frequency relaxation mode in the spectrum. In principle, the lower-frequency modes observed for the present solutions could at least partly arise from such “slow water.” However, evaluation of the amplitudes of these processes as a function of solute concentration yielded results that were neither self-consistent nor compatible with previous findings.^7,33,34^ Accordingly, the present total hydration numbers were exclusively assigned to irrotationally bound H_2_O dipoles, i.e., *Z*_t_ = *Z*_ib_.

Detailed analysis of the DR spectra of simple nitrate salts such as NaNO_3_ in aqueous solution,^35^ as well as information from double-difference-IR spectroscopy,^36^ 2D-IR spectroscopy,^37,38^ and *ab initio* MD simulations^39^ indicate that the nitrate ion does not slow down but accelerates the dynamics of the ∼6 to 9 H_2_O molecules^40^ in its hydration shell. Obviously, the associated relaxation time does not differ sufficiently from that of bulk water to allow resolution of this contribution as a separate mode in the present DR spectra. The increasing amount of this fast hydration water is almost certainly the main reason for the observed decrease of τ_2_ with rising *c* (Figure S6). Similar to the situation for Cl^–^,^41^ we therefore reasonably assume *Z*_t_(NO_3_^–^) = 0 in the following analysis.

Accordingly, the effective hydration numbers shown in Figure 5 can be assigned fully to the cations. In line with their surface-charge densities, *Z*_t_(M(NO_3_)_*z*_) = *Z*_ib_(M(NO_3_)_*z*_) = *Z*_ib_(M^*z*+^) increases in the order Tl^+^ < Ag^+^ < Pb^2+^ < Cd^2+^. While the *Z*_t_ values for Tl^+^ and Pb^2+^ decrease linearly with rising *c*, those for Ag^+^ and Cd^2+^ exhibit an exponential decay, apparently leveling off respectively at *a*_1_ ≈ 1.3 and ∼11.6 at high *c*, (Table 1). However, for none of the present salts does the concentration dependence of *Z*_t_(*c*) exhibit a discontinuity at the concentration where (due to crowding) the anion starts to penetrate the second hydration shell of the cation (Ag^+^: ∼1.29 M; Tl^+^: ∼1.14 M; Cd^2+^: ∼0.92 M; Pb^2+^: ∼0.85 M).^42^

**Table 1:** Parameters *Z*_t_^0^, *a*_1_, and *a*_2_ Describing the Concentration Dependence of the Effective Total Cation Hydration Numbers, *Z*_t_, of the Studied Metal Nitrates, and First-Shell Cation Coordination Numbers, CN_+_, from the Literature^a^,^b^

	*Z*_t_^0^	*a*_1_	*a*_2_	CN_+_^(1)^
TlNO_3_^c^	4.4 ± 0.6		6.8 ± 2.2	5.9^9^
AgNO_3_^d^	5.9 ± 0.3	1.3 ± 0.1	0.66 ± 0.06	2 + 3 and 2 + 4;^43^ 2 + *x*^44^
Pb(NO_3_)_2_^c^	9.7 ± 0.4		3.5 ± 0.4	8.1;^45^ 6;^11^ 6–9^46^
Cd(NO_3_)_2_^d^	20.5 ± 1.2	11.6 ± 0.5	0.85 ± 0.13	6–7^10,40,47^

a Based on the assumption *Z*_t_(NO_3_^–^) = 0, see text for details.

b Units: *a*_2_ in M^–1^.

c *Z*_t_ = *Z*_t_^0^ – *a*_2_ × *c*.

d *Z*_t_ = *a*_1_ + (*Z*_t_^0^ – *a*_1_) × exp(−*c*/*a*_2_).

**Figure 6:**
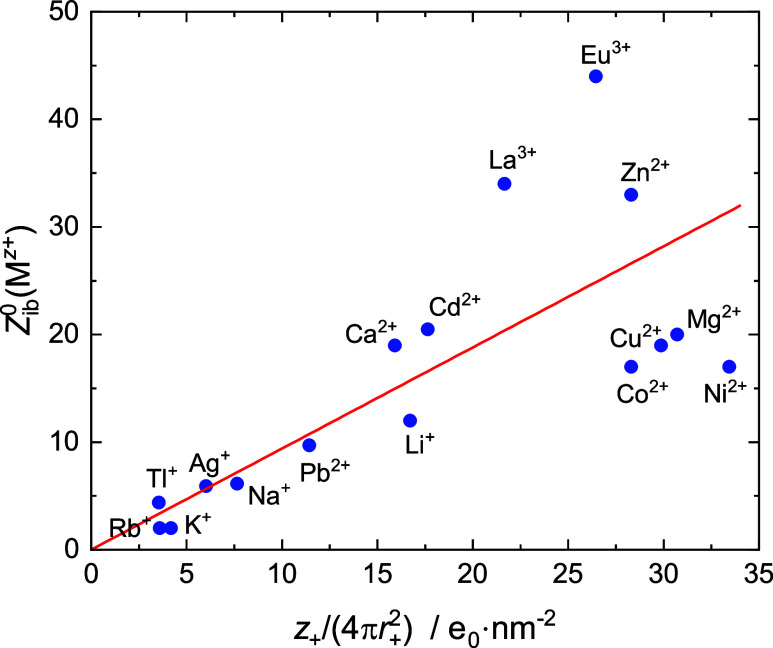
Number, *Z*_ib_^0^(M^*z*+^), of H_2_O dipoles frozen (irrotationally bound) by the cation at *c* → 0 as a function of its surface-charge density, *z*_+_*e*_0_/(4π*r*_+_^2^) (*e*_0_ is the elementary charge), for the present cations and literature data.^33,41,48−51^ Note that *Z*_ib_^0^(M^*z*+^) = *Z*_t_^0^(M^*z*+^), except for K^+^ and Rb^+^, where *Z*_ib_^0^ < *Z*_t_^0^.^41^ The line is a linear fit of all data forced through the origin.

Ion–ion interactions, which can lead to a “softening” or breakup of ion hydration shells, are negligible as *c* → 0. Accordingly, the intercepts *Z*_t_^0^(M^*z*+^) reflect the strength of cation-water interactions. For those, the electrostatic force between cation charge and H_2_O dipole moment, and thus the surface-charge density of the ion, *z*_+_*e*_0_/(4π*r*_+_^2^) (*e*_0_ is the elementary charge), is commonly assumed to be the dominant factor.^52^Figure 6 shows that *Z*_t_^0^(M^*z*+^) indeed correlates reasonably with cation surface-charge density, although increasing deviations at high *Z*_t_^0^ and/or *z*_+_*e*_0_/(4π*r*_+_^2^) values are obvious.

The intercepts *Z*_t_^0^(M^*z*+^) can also be compared with cation coordination numbers, CN_+_, derived from computer simulations or scattering experiments.^7^ Generally, such data are only available for the first hydration shell (CN_+_^(1)^) but in some cases also the number of second-nearest neighbors, CN_+_^(2)^, was determined.^40^ According to the data for Ag^+^^43,44^ and Pb^2+^,^11,45,46^*Z*_t_^0^ ≈ CN_+_^(1)^ (Table 1). This means that the dynamics of the H_2_O molecules residing in the second hydration shell of these ions (∼5 to 17^8,40^ for Ag^+^ and ∼12^11^ for Pb^2+^) are too similar to bulk water to be resolved by DRS. In contrast, H_2_O dipoles in direct contact with Tl^+^ are apparently not completely frozen (Table 1), in line with results from QM/MM-MD simulations,^9^ revealing a somewhat dynamic hydration shell for this ion. In dilute aqueous solutions the first hydration shell of Cd^2+^ contains 6–7 H_2_O molecules^10,40,47^ whereas ∼14 to 16 H_2_O molecules are in the second shell.^10^ Thus, the present value of *Z*_t_^0^(Cd^2+^) = 20.5 ± 1.2 (Table 1) indicates that as *c* → 0 the water dipoles in the second hydration shell become essentially frozen. As *c* increases, *Z*_t_ → 11.6 > CN_+_^(1)^. This suggests that the marked nonlinear decrease of *Z*_t_(*c*) with increasing *c* for Cd^2+^(aq) (Figure 5) mainly reflects second-hydration shell breakup due to ion–ion interactions, similar to results reported for other divalent cations such as Mg^2+^, Ca^2+^, Cu^2+^, Co^2+^ or Ni^2+^.^33,48,49^ For Ag^+^(aq) one may speculate that the drop of *Z*_t_(*c*) from *Z*_t_^0^ ≈ 5.9 to ∼1.3 reflects the unusual solvation shell structure of this ion: a well-defined (thus long-lived) linear [Ag(H_2_O)_2_]^+^ moiety with a few more mobile perpendicular H_2_O dipoles at slightly longer Ag–O distances.^44^

**Figure 7:**
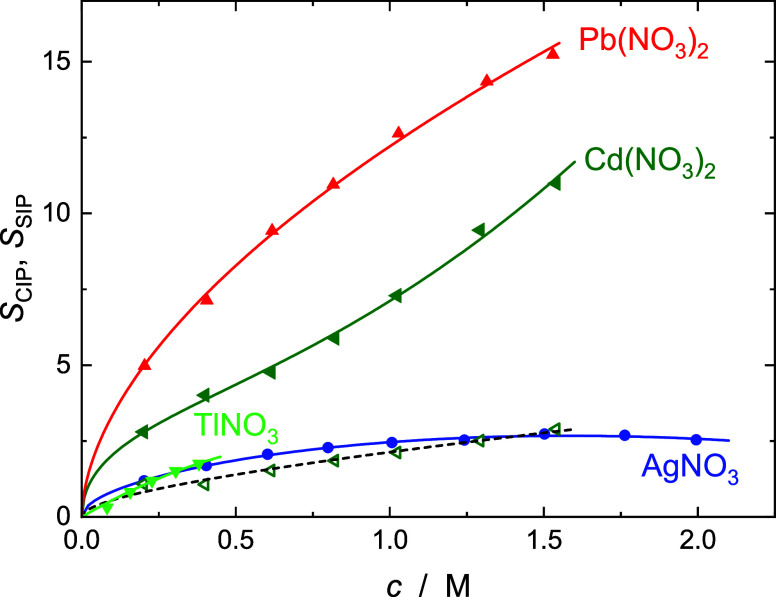
Contact-ion-pair amplitudes, *S*_CIP_ (filled symbols), and their fits (solid lines), for aqueous solutions of: AgNO_3_ (blue ○), TlNO_3_ (green ▽), Cd(NO_3_)_2_ (olive ◁), and Pb(NO_3_)_2_ (red △) as functions of solute concentration, *c*, at 25 °C. Also included is the amplitude, *S*_SIP_ (open symbols, broken line), assigned to solvent-shared [Cd(OH_2_)NO_3_]^+^(aq) ion pairs.

The influence of the dissolved ions is not restricted to their immediate hydration shell(s) but also affects bulk-water dynamics. For the present salts the relaxation time associated with the cooperative resettling of the H-bond network, τ_CC_ (=τ_2_ for the D+CC+D model; =τ_3_ for the D+D+CC+D model), exhibits a significant initial decrease with increasing *c* before leveling at *c* ≳ 0.7 M (Figure S6). Simultaneously, the associated peak-width parameter, α_CC_, rises monotonically (Figure 7), indicating increasing microheterogeneity of the solvent molecules in the solutions. The increasing concentration of nitrate ions is one reason for the drop of τ_CC_(*c*), because the orientational relaxation time of H_2_O molecules in the immediate vicinity of the anion reaches only ∼60% of the bulk water value.^39^ However, the cations also have an effect since the decrease of τ_CC_(*c*) follows the surface-charge density of the cation (Figure S6). This may indicate an increasing mismatch between the structures of bulk- and hydration-shell water, reducing the number of H-bonds between them. Indeed, for all studied cations simulations suggest rather mobile second hydration shells with frequent exchange of H_2_O molecules with the bulk.^9,44,45,47^ Also, results from DRS,^53,54^ Raman,^54^ and terahertz spectroscopy^28^ suggest the existence of such a region of hypermobile water around many ions.

### Solute Relaxation(s)

All the present electrolytes exhibited a low-frequency mode centered at ∼1.5 to 3.0 GHz (Figures 2 and 3). From the corresponding relaxation times of ca. ∼50 to 100 ps (Tables S1–S4) the most plausible origins of this mode are either dynamically retarded (but not frozen) cation hydration-shell water or the formation of contact ion pairs (CIPs). As indicated in the previous section, the amplitudes of this mode (Figure 7) yielded “slow water” hydration numbers, *Z*_s_, greater than the total amount of bound water, *Z*_t_ (Figure 5), which is physically impossible. On the other hand, there is convincing evidence in the literature that all four cations form weak (or very weak) ion pairs with nitrate in aqueous solution.^55−62^ Accordingly, and consistent with its location in the spectra, this relaxation mode is attributed to CIPs for AgNO_3_, TlNO_3_, Cd(NO_3_)_2_ and Pb(NO_3_)_2_. The additional lowest-frequency mode resolved for the cadmium salt centered at ∼0.6 GHz (Figure 3) is assigned to solvent-shared ion pairs (SIPs), [Cd(OH_2_)NO_3_]^+^(aq), in line with the strong hydration of Cd^2+^ (see above).

Effective ion-pair dipole moments, μ_eff,IP_ = μ_IP_/(1 – *f*_IP_α_IP_), reaction-field, *f*_IP_, and cavity-field factors, *A*_IP_, required to calculate ion-pair concentrations, *c*_IP_, via eq 3 using the ion-pair amplitudes, *S*_CIP_ and *S*_SIP_ of Figure 7, were obtained following the procedure of Barthel et al.^63^ Radii, *r*_*i*_, and electronic polarizabilities, α_*i*_, of cations (*i* = +), anions (−) and solvent (*w*) are summarized in Table S5.^32^ The polarizability of the ion pair α_IP_ was estimated as α_+_ + α_–_ + *mα*_w_, with *m* = 0 for CIPs and 1 for SIPs. The center of hydrodynamic stress was taken to be the pivot for the charged [PbNO_3_]^+^(aq) and [Cd(OH_2_)_*m*_NO_3_]^+^(aq) ion pairs.^64^ The resulting permanent (vacuum) ion-pair dipole moments, μ_IP_, are included in Table 2.

**Table 2:** Ion-Pair Dipole Moments, μ_IP_; Parameters *K*_*i*_^0^ (*i* = A or SIP), *B*, and *C* of eq 9 or *K*_CIP_^0^ and *B* for the Linear Fit of *K*_CIP_; and *K*_A_^0^ Values from the Literature^a^

	IP	μ_IP_	*K*_A_^0^	*B*	*C*	*K*_A_^0^(lit)
AgNO_3_	CIP	12.58	4.6 ± 0.7	–2.26 ± 0.23	0.98 ± 0.15	3.7^b^
TlNO_3_	CIP	13.81	2.38 ± 0.13	–1.40 ± 0.09		2.3,^b^ 3.2^56^
Pb(NO_3_)_2_	CIP	18.96	6.6 ± 0.9	–0.62 ± 0.08	0.18 ± 0.03	see text
Cd(NO_3_)_2_	SIP+CIP		8.7 ± 2.8^c^	–0.84 ± 0.03	0.28 ± 0.02	see text
	SIP	45.69	0.76 ± 0.23	–0.87 ± 0.18	0.29 ± 0.08	
	CIP	20.30	10.4 ± 1.1	0.8 ± 0.4^d^		

a Units: μ_IP_ in D; *K*_A_^0^, *K*_SIP_^0^, *K*_A_^0^(lit) in M^–1^; *B* in M^–1^; *C* in M^–3/2^.

b Values of Tsierkezos and Ritter^61^ interpolated to 298.15 K.

c Fixed to *K*_A_^0^ = *K*_SIP_^0^ + *K*_SIP_^0^*K*_CIP_^0^.

d Linear fit *K*_SIP_ = *K*_SIP_^0^ + *B* × *I*.

**Figure 8:**
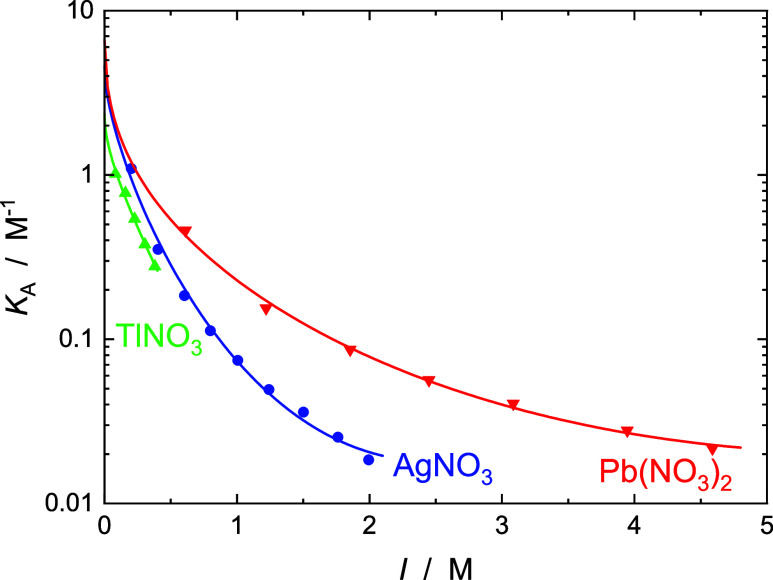
Association constants, *K*_A_ (symbols), for aqueous solutions of AgNO_3_, TlNO_3_, and Pb(NO_3_)_2_ as a function of ionic strength, *I*, at 25 °C, assuming only CIP formation. Lines are fits with eq 9 (see Table 2).

**Figure 9:**
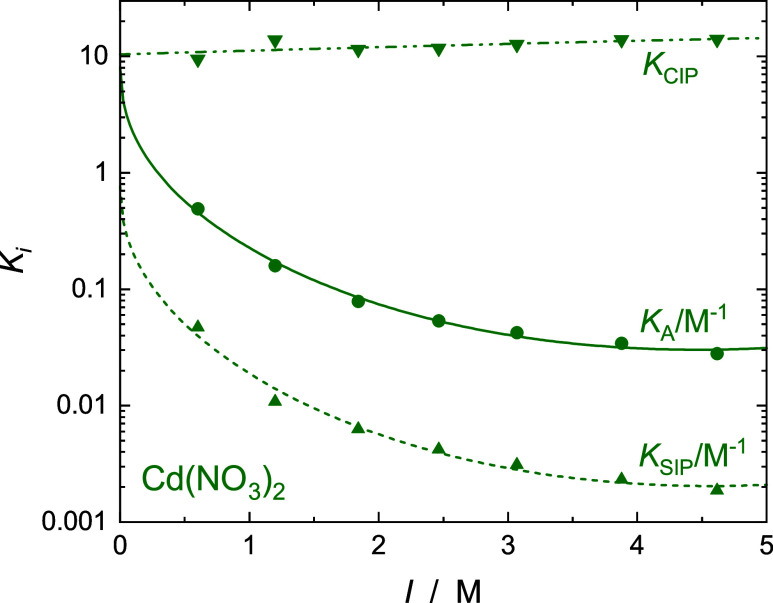
Overall *K*_A_ (•), and stepwise association constants for SIP, *K*_SIP_ (▲), and for CIP, *K*_CIP_ (▼), in aqueous solutions of Cd(NO_3_)_2_ as a function of ionic strength, *I*, at 25 °C. Lines are fits with eq 9 for *K*_A_ and *K*_SIP_, or a linear fit for *K*_CIP_ (see data in Table 2).

Overall association constants, *K*_A_ = *c*_IP_/(*c*_+_*c*_–_), calculated from the total ion-pair concentrations, *c*_IP_, for TlNO_3_(aq), AgNO_3_(aq), and Pb(NO_3_)_2_(aq), where only CIPs were detected, are shown as a function of ionic strength in Figure 8. Note that under these conditions *c*_IP_ = *c*_CIP_, *c*_+_ = *c* – *c*_IP_ and *c*_–_ = ν_–_*c* – *c*_IP_ (where ν_–_ = 1 or 2 is the stoichiometric coefficient of the anion). The equivalent data for Cd(NO_3_)_2_(aq), where *c*_IP_ = *c*_CIP_ + *c*_SIP_, as well as the stepwise association constants, *K*_SIP_ = *c*_SIP_/(*c*_+_*c*_–_) and *K*_CIP_ = *c*_CIP_/*c*_SIP_, corresponding to the equilibria
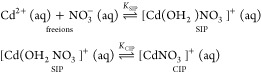
8are shown in Figure 9.

For all four studied electrolytes, *K*_A_ values show the typical decrease with rising nominal ionic strength, *I* = 0.5 × ∑ *c*_*i*_*z*_*i*_^2^ (*i* = +, −), that can be described for convenience by an extended Guggenheim-type equation^34,65^
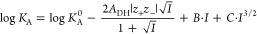
9where *K*_A_^0^ is the standard state (infinite dilution) association constant, *z*_+_ and *z*_–_ are the ion charge numbers, *A*_DH_ = 0.5115(L mol^–1^)^1/2^ is the Debye–Hückel constant for activity coefficients in water at 25 °C, and *B* and *C* are adjustable parameters. Equation 9 also describes the ionic-strength dependence of *K*_SIP_ for Cd(NO_3_)_2_(aq), whereas log*K*_CIP_ exhibits a weak linear increase (Figure 9). The corresponding fit parameters are summarized in Table 2.

All the present nitrate salts exhibit weak to very weak ion association, with *K*_A_ values at any given ionic strength (including infinite dilution, Table 2), following the order Tl^+^ < Ag^+^ < Pb^2+^ < Cd^2+^ (Figures 8 and 9) consistent with the charge/radius ratio of the cations. Perhaps more significantly, given that the association reaction involves the replacement of one or more water molecules in the hydration shell(s) of the cation by the incoming NO_3_^–^ ion, the above sequence is also consistent with their (absolute) hydration energies (−Δ_hyd_*G*° = 310, 440, 1434, and 1736 kJ·mol^–1^, respectively).^32^

The *K*_A_^0^ values obtained via eq 9 can be compared directly with literature results. For convenience, comparisons with the present results are made with constants taken from the comprehensive IUPAC compilations,^66−68^ supplemented where appropriate with more recent publications. However, before attempting such comparisons, some words of warning are appropriate. In general, the reliable measurement of ion-association constants for which *K*_A_^0^ ≲ 5 (or even ≲10) M^–1^ is extraordinarily difficult (some would say impossible). Some techniques are undoubtedly better than others for the quantification of weak complexes and it is sometimes possible to create favorable circumstances for their measurement.^69,70^ Nevertheless, overall the generalization remains valid and it is wise to view all small values of *K*_A_ with caution. This unhappy situation is apparent from the rather large uncertainties in the present *K*_A_^0^ values, and from the lack of agreement among the literature results, even when no particular problem can be identified.

Despite the concerns just expressed, the agreement between the present and selected literature values of *K*_A_^0^ for AgNO_3_^0^(aq) and TlNO_3_^0^(aq) is excellent (Table 2). Note, however, that there are other plausible literature results^66−68^ that are more scattered.

The situation regarding the formation of PbNO_3_^+^(aq) is less satisfactory. Several older but apparently reliable investigations using traditional techniques are in good agreement with each other and suggest *K*_A_^0^ ≈ 14 M^–1^. However, others cluster around *K*_A_^0^ ≈ 3 M^–1^.^66−68^ More recent results using ^207^Pb-NMR^62,71^ are lower still but are almost certainly incorrect.^72^ On the other hand, Raman spectroscopy appears to tell a different story.^60^ Thus, although Xu et al. did not derive equilibrium constants,^60^ the CIP concentrations that can be abstracted from their spectra at *c* ≥ 1 M are about five-times greater than the present results under comparable conditions. Apart from the different time-scales of Raman and DR spectroscopies, with the former being sensitive to short-lived (<ps) ion contacts while the latter requires an IP lifetime comparable to τ_CIP_, there is no obvious explanation of these discrepancies. One possibility might be the formation of pseudolinear ion triplets, [O_3_N Pb NO_3_]^0^(aq), analogous to those found for M(ClO_4_)_2_ in acetonitrile.^73^ Such species are Raman-active but do not have a DRS signature. All that can be said is that the present result: *K*_A_^0^ ≈ 7 M^–1^ (Table 2) sits in the middle of these disparate values.

The formation constant for CdNO_3_^+^(aq) has been less well reported in the literature. Based on the systematic behavior noted above, the present DRS results: *K*_A_^0^(CdNO_3_^+^) > *K*_A_^0^(PbNO_3_^+^) would seem to be more realistic than the opposite suggested by the sparse and uncertain literature values.^66−68^

As noted in the Introduction, a great advantage of DRS is its ability to identify and quantify the types of ion pairs present, i.e., the extent to which they exist as CIPs, SIPs or 2SIPs.^7^ The DR spectra for the nitrate solutions of Ag^+^, Tl^+^ and Pb^2+^ identify, from their location in the frequency domain, the presence only of CIPs. For the more strongly hydrated Cd^2+^, SIPs are also formed (albeit in only small amounts). In this context it is interesting to note that ^207^Pb-NMR spectra^62,71^ also indicate the presence of CIPs in Pb(NO_3_)_2_(aq) solutions. Raman measurements^60,74^ on various divalent nitrate solutions show that stronger cation hydration results in increasing formation of SIPs, as found here for Cd(NO_3_)_2_(aq) solutions.

## Conclusions

Broadband dielectric relaxation spectra of the aqueous solutions of four heavy-metal nitrate salts (TlNO_3_, AgNO_3_, Pb(NO_3_)_2_ and Cd(NO_3_)_2_) at 25 °C reveal the presence of three or (for the Cd salt only) four modes: one (two for Cd) solute-related mode(s) at lower frequencies and two solvent-related modes at higher frequencies. Detailed analysis of the latter indicated that all four cations were strongly hydrated with effective hydration numbers (*Z*_t_ values) of approximately 4, 6, 10, and 20 irrotationally bound water molecules at infinite dilution for Tl^+^, Ag^+^, Pb^2+^ and Cd^2+^, respectively, consistent with the partial retardation of H_2_O molecules in the second hydration shell for Pb^2+^(aq) and especially for Cd^2+^(aq). The solute-related mode(s) were consistent with the rotational diffusion of contact ion pairs in all four systems, with small amounts of solvent-shared IPs for the more strongly hydrated Cd^2+^(aq). The present overall ion-pair formation constants are in good agreement with literature values for TlNO_3_^0^(aq) and AgNO_3_^0^(aq). However, the agreement is less satisfactory for PbNO_3_^+^(aq) and CdNO_3_^+^(aq). Such differences are at least partly due to inadequacies in the literature data. There is a clear need for careful redetermination of these constants using reliable techniques such as metal amalgam potentiometry.

## Supporting Information Available

Tables with experimental densities, conductivities and dielectric relaxation parameters of the investigated solutions; additional figures supporting data processing and discussion (PDF)
