# Supplementary material for: Ion Association and Hydration of Some Heavy-Metal Nitrate Salts in Aqueous Solution
Source: J Phys Chem B. 2024 Oct 3;128(41):10238–46. doi: 10.1021/acs.jpcb.4c05441 (PMC11492267; doi:10.1021/acs.jpcb.4c05441)
Supplement: Supplementary file 1 — jp4c05441_si_001.pdf [file jp4c05441_si_001.pdf]

Supporting Information: Ion Association and Hydration of Some Heavy-Metal Nitrate Salts in Aqueous Solution

Johannes Hunger,*,† Richard Buchner,*,‡ and Glenn Hefter*,¶

†Department for Molecular Spectroscopy, Max Planck Institute for Polymer Research, D-55128 Mainz, Germany

‡Institut für Physikalische und Theoretische Chemie, Universität Regensburg, D-93040 Regensburg, Germany

¶Chemistry Department, Murdoch University, Murdoch, WA 6150, Australia

E-mail: hunger@mpip-mainz.mpg.de; richard.buchner@chemie.uni-regensburg.de; g.hefter@murdoch.edu.au

Spectra and their fits

Table S1: Density, ρ, electrical conductivity, κ, and fit parameters (static permittivity, ε, amplitudes, S_j, and relaxation times, τ_j, and Cole-Cole width parameter α_2 of the resolved modes, $j = 1\ldots3$) of the D+CC+D model for the dielectric spectra of $AgNO_3$(aq) at 25 °C and concentration, c. For all samples relaxation time τ_3 and infinite frequency permittivity ε_∞ were set to the values of neat water, 0.278 ps and 3.52.[S1] [a]

c	ρ	κ	ε	S_1	τ_1	S_2	τ_2	α_2	S_3
0.203	1.0254	1.96	76.85	1.19	138	69.97	7.93	0.002	2.17
0.404	1.0539	3.54	75.59	1.69	85.5	68.13	7.82	0.003	2.25
0.604	1.0808	4.95	74.55	2.06	74.4	66.62	7.75	0.006	2.35
0.799	1.1075	6.18	73.67	2.28	65.2	65.55	7.70	0.009	2.32
1.007	1.1360	7.39	72.82	2.45	58.5	64.46	7.68	0.012	2.39
1.242	1.1689	8.66	71.85	2.53	53.1	63.40	7.65	0.017	2.40
1.502	1.2059	9.93	70.96	2.73	48.9	62.24	7.65	0.020	2.47
1.763	1.2421	11.10	70.19	2.69	50.5	61.51	7.66	0.026	2.47
1.994	1.2735	12.09	69.56	2.54	54.9	61.07	7.69	0.032	2.44

[a] Units: c in M, ρ in kg L^{-1}, κ in S m^{-1}, τ_j in ps.

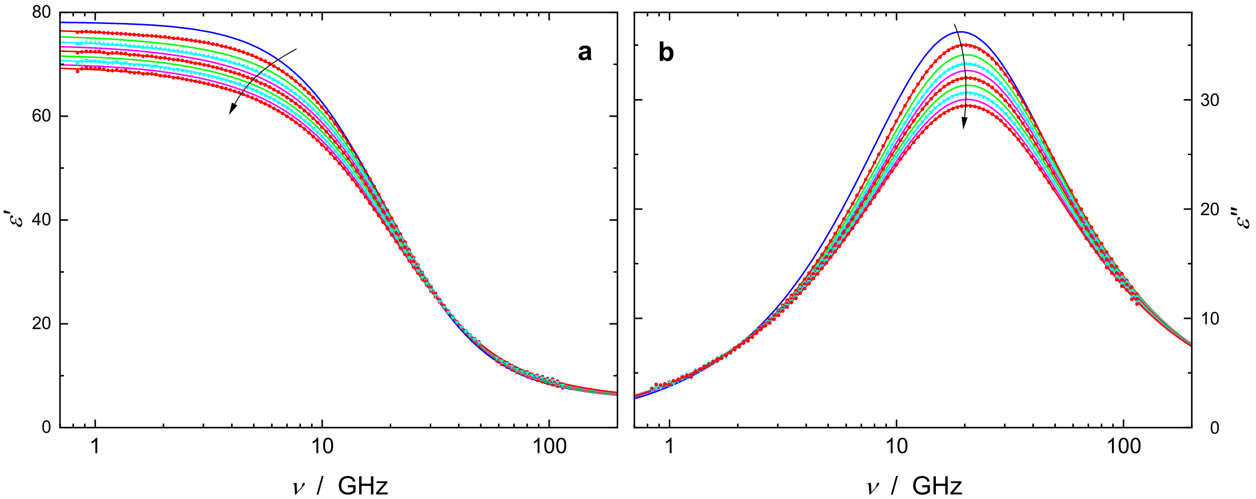

Figure S1: (**a**) Relative permittivity, $\varepsilon'(\nu)$, and (**b**) dielectric loss, $\varepsilon''(\nu)$, spectra of aqueous solutions of $AgNO_3$ at 25 °C (symbols) and their fits with the D+CC+D model (lines). Arrows indicate increasing solute concentrations, c / M = 0.204, 0.406, 0.604, 0.799, 1.007, 1.242, 1.502, 1.736, 1.994. Experimental points have been partly omitted for visual clarity. For comparison, the spectrum of neat water (c / M = 0) calculated from the relaxation parameters of Eiberweiser et al.[S1] (S_1 = 72.42, τ_1 = 8.35ps, S_2 = 2.43, τ_2 = 0.278ps, ε_∞ = 3.52) is also shown.

Table S2: Density, ρ, electrical conductivity, κ, and fit parameters (static permittivity, ε, amplitudes, S_j, and relaxation times, τ_j, and Cole-Cole width parameter α_2 of the resolved modes, $j = 1 \ldots 3$) of the D+CC+D model for the dielectric spectra of $TlNO_3$(aq) at 25 °C and concentration, c. For all samples relaxation time τ_3 and infinite frequency permittivity ε_∞ were set to the values of neat water, 0.278 ps and 3.52.[S1] [a]

c	ρ	κ	ε	S_1	τ_1	S_2	τ_2	α_2	S_3
0.082	1.0157	0.97	77.47	0.31	73.9	71.14	8.11	0.0002	2.49
0.158	1.0326	1.69	77.33	0.82	85.7	70.50	8.08	0.0010	2.49
0.228	1.0485	2.31	76.79	1.19	89.1	69.54	8.01	0.0024	2.54
0.305	1.0659	2.96	76.73	1.51	79.0	69.11	7.94	0.0029	2.58
0.381	1.0832	3.57	76.59	1.75	69.3	68.69	7.87	0.0036	2.62

[a] Units: c in M, ρ in kg L^{-1}, κ in S m^{-1}, τ_j in ps.

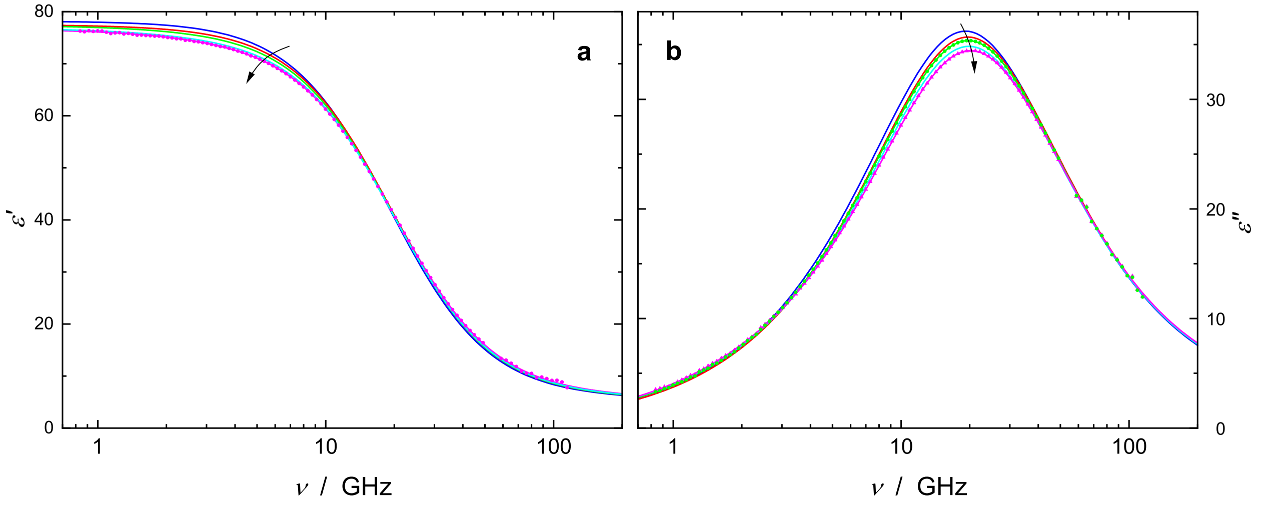

Figure S2: (**a**) Relative permittivity, $\varepsilon'(\nu)$, and (**b**) dielectric loss, $\varepsilon''(\nu)$, spectra of aqueous solutions of $TlNO_3$ at 25 °C (symbols) and their fits with the D+CC+D model (lines). Arrows indicate increasing solute concentrations, c / M = 0.082, 0.158, 0.228, 0.305, 0.381. Experimental points have been partly omitted for visual clarity. For comparison, the spectrum of neat water (c / M = 0) calculated from the relaxation parameters of Eiberweiser et al.[S1] (S_1 = 72.42, τ_1 = 8.35ps, S_2 = 2.43, τ_2 = 0.278ps, ε_∞ = 3.52) is also shown.

Table S3: Density, ρ, electrical conductivity, κ, and fit parameters (static permittivity, ε, amplitudes, S_j, and relaxation times, τ_j, and Cole-Cole width parameter α_2 of the resolved modes, $j = 1 \ldots 3$) of the D+CC+D model for the dielectric spectra of $Pb(NO_3)_2$(aq) at 25 °C and concentration, c. For all samples relaxation time τ_3 and infinite frequency permittivity ε_∞ were set to the values of neat water, 0.278 ps and 3.52.[S1] [a]

c	ρ	κ	ε	S_1	τ_1	S_2	τ_2	α_2	S_3
0.204	1.0554	2.65	79.01	4.98	106	68.37	7.78	0.012	2.14
0.406	1.1133	4.21	78.41	7.13	74.9	65.28	7.75	0.019	2.49
0.618	1.1745	5.44	77.88	9.43	63.6	62.14	7.59	0.025	2.79
0.816	1.2258	6.25	77.90	10.95	63.7	60.63	7.56	0.036	2.80
1.029	1.2877	7.07	77.73	12.63	64.0	58.72	7.45	0.047	2.86
1.315	1.3772	7.86	77.38	14.35	65.4	56.50	7.49	0.065	3.01
1.529	1.4217	8.17	77.06	15.23	66.0	55.17	7.51	0.072	3.14

[a] Units: c in M, ρ in kg L^{-1}, κ in S m^{-1}, τ_j in ps.

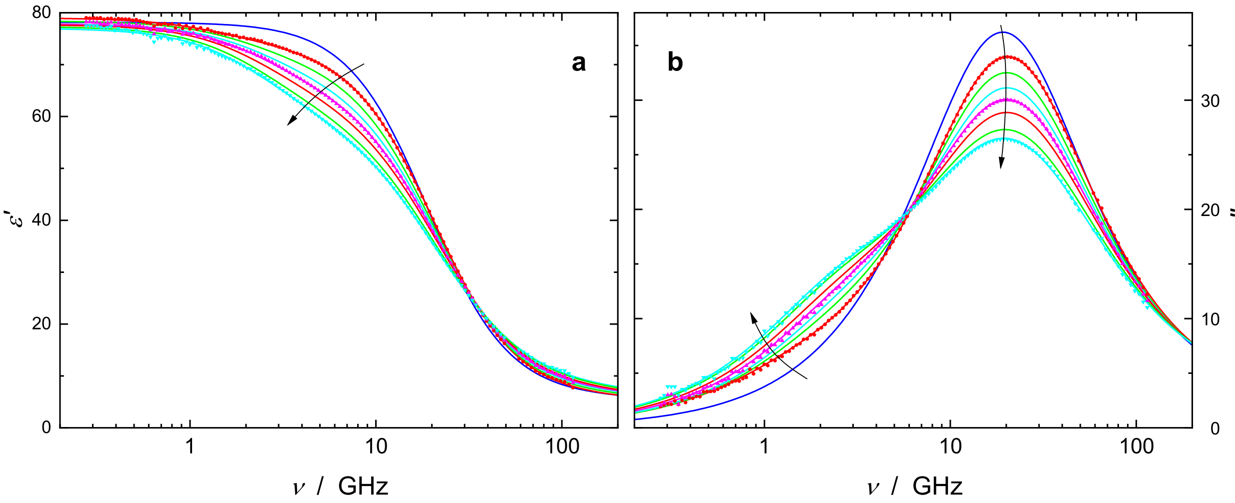

Figure S3: (**a**) Relative permittivity, $\varepsilon'(\nu)$, and (**b**) dielectric loss, $\varepsilon''(\nu)$, spectra of aqueous solutions of $Pb(NO_3)_2$ at 25 °C (symbols) and their fits with the D+CC+D model (lines). Arrows indicate increasing solute concentrations, c / M = 0.204, 0.406, 0.618, 0.816, 1.029, 1.315, 1.529. Experimental points have been partly omitted for visual clarity. For comparison, the spectrum of neat water (c / M = 0) calculated from the relaxation parameters of Eiberweiser et al.[S1] (S_1 = 72.42, τ_1 = 8.35ps, S_2 = 2.43, τ_2 = 0.278ps, ε_∞ = 3.52) is also shown.

Table S4: Density, ρ, electrical conductivity, κ, and fit parameters (static permittivity, ε, amplitudes, S_j, and relaxation times, τ_j, and Cole-Cole width parameter α_3 of the resolved modes, $j = 1 \ldots 4$) of the D+D+CC+D model for the dielectric spectra of $Cd(NO_3)_2$(aq) at 25 °C and concentration, c. For all samples relaxation time τ_4 and infinite frequency permittivity ε_∞ were set to the values of neat water, 0.278 ps and 3.52.[S1] [a]

c	ρ	κ	ε	S_1	τ_1	S_2	τ_2	S_3	τ_3	α_3	S_4
0.201	1.0377	2.96	75.59	1.09	272	2.80	117	65.90	7.71	0.002	2.28
0.400	1.0772	5.12	72.09	1.07	260	4.01	100	61.05	7.45	0.007	2.44
0.614	1.1219	6.92	69.14	1.53	259	4.77	96.8	56.71	7.33	0.014	2.61
0.821	1.1643	8.22	67.16	1.85	267	5.89	103	53.25	7.21	0.023	2.65
1.023	1.2037	9.08	66.07	2.12	286	7.29	112	50.44	7.13	0.032	2.69
1.293	1.2578	9.83	64.98	2.51	282	9.45	117	46.64	6.99	0.040	2.86
1.539	1.3052	10.12	64.32	2.89	266	10.99	121	43.87	7.07	0.052	3.05

[a] Units: c in M, ρ in kg L^{-1}, κ in S m^{-1}, τ_j in ps.

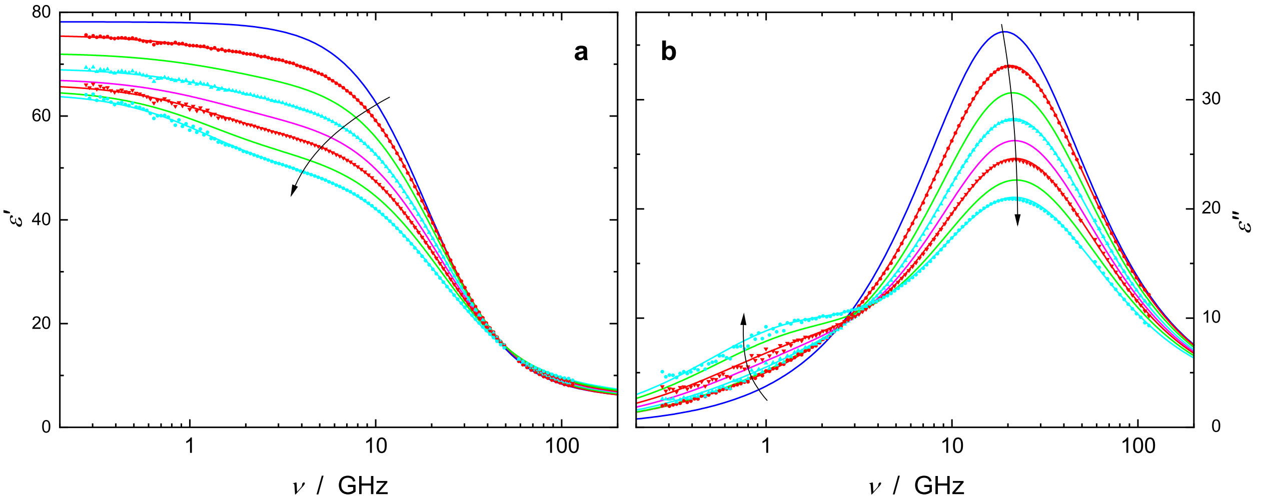

Figure S4: (**a**) Relative permittivity, $\varepsilon'(\nu)$, and (**b**) dielectric loss, $\varepsilon''(\nu)$, spectra of aqueous solutions of $Cd(NO_3)_2$ at 25 °C (symbols) and their fits with the D+D+CC+D model (lines). Arrows indicate increasing solute concentrations, c / M = 0.201, 0.400, 0.614, 0.821, 1.023, 1.293, 1.539. Experimental points have been partly omitted for visual clarity. For comparison, the spectrum of neat water (c / M = 0) calculated from the relaxation parameters of Eiberweiser et al.[S1] (S_1 = 72.42, τ_1 = 8.35ps, S_2 = 2.43, τ_2 = 0.278ps, ε_∞ = 3.52) is also shown.

Table S5: Radii, r_i, and electronic polarizabilities, α_i, of solvent and ions.[a,b]

	H_2O	NO_3^-	Tl^+	Ag^+	Pb^{2+}	Cd^{2+}
r_i	142.5	179	150	115	118	95
α_i	1.444	4.12	4.56	2.02	4.72	1.28

[a] Units: r_i in pm; α_i in $10^{-30}m^3$. [b] Ref. S2

Solvent relaxation

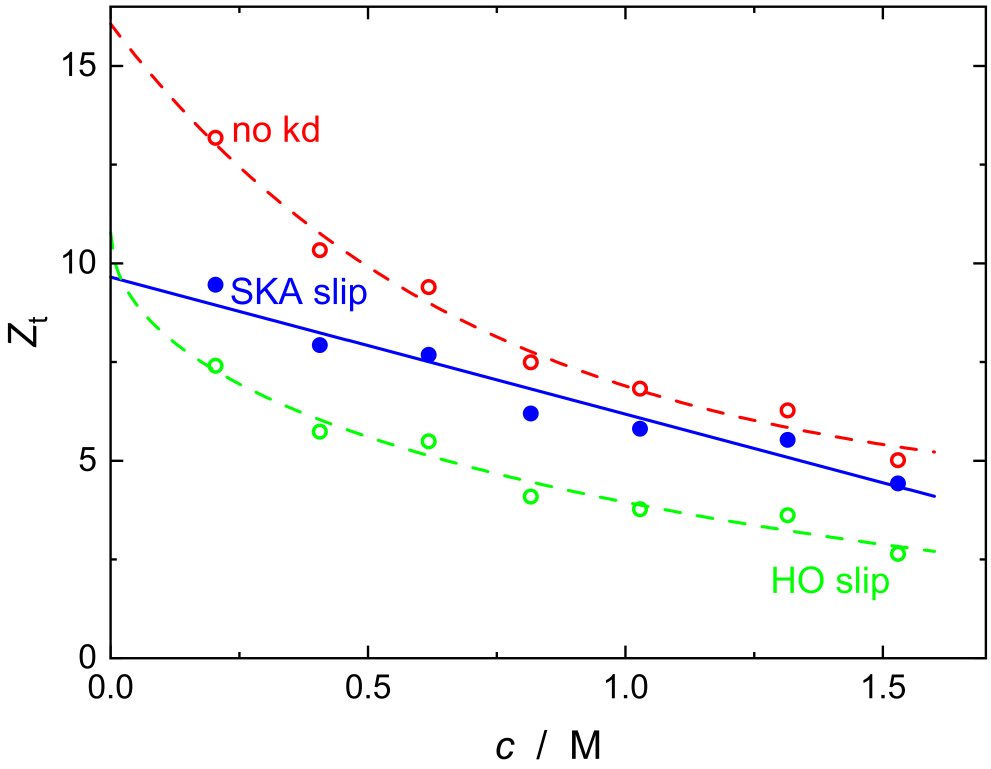

Figure S5: Total effective hydration numbers, Z_t, of $Pb(NO_3)_2$ as a function of solute concentration calculated from the corresponding bulk-water amplitude, $S_b = S_2 + S_3$ (Table S3), assuming no kinetic depolarization (”no kd”, red circles), kd with slip boundary conditions according to the corrected Hubbard-Onsager (HO) approach[S3] (green circles), or after its semi-empirical extension by Sega, Kantorovich and Arnold (SKA, blue filled circles).[S4] Lines are empirical fits as a guide to the eye.

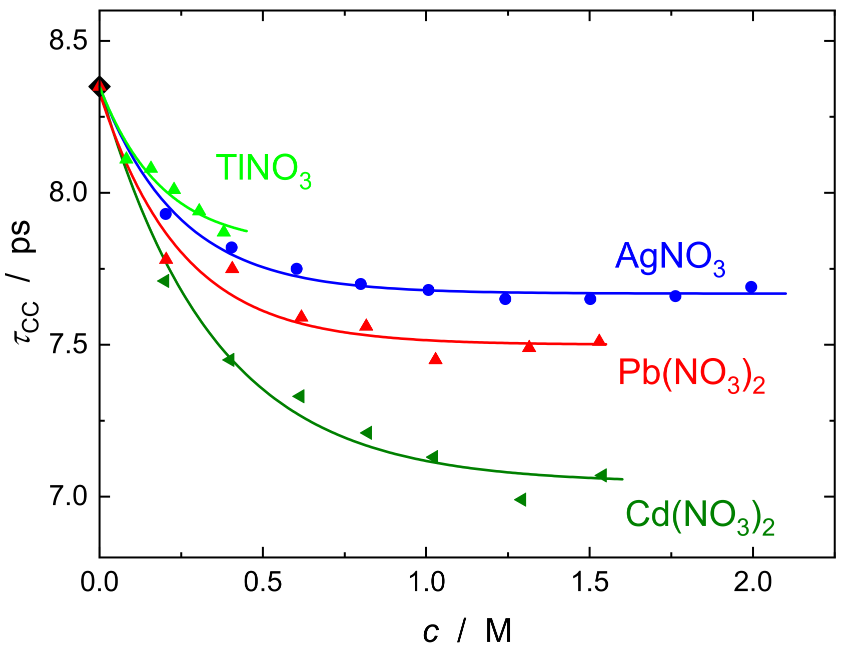

Figure S6: Dielectric relaxation time of the Cole-Cole mode, τ_{CC} (symbols), representing the cooperative H-bond-network rearrangement of water, as a function of solute concentration, c, for the investigated metal nitrate solutions. Lines are empirical fits as a guide to the eye.

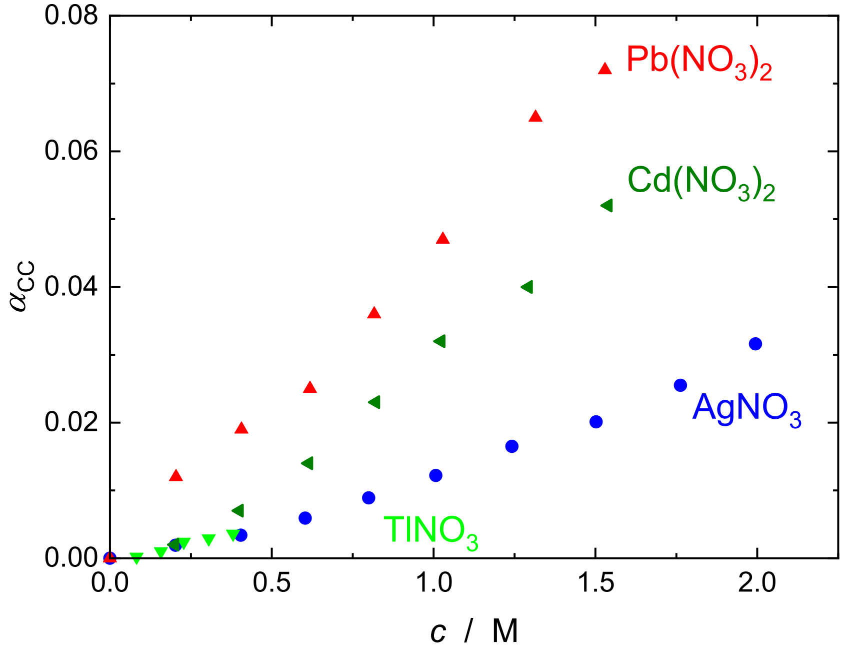

Figure S7: Cole-Cole width parameter, α_{CC} (symbols), of the cooperative H-bond-network rearrangement of water, as a function of solute concentration, c, for the investigated metal nitrate solutions.

References

(S1) Eiberweiser, A.; Nazet, A.; Hefter, G.; Buchner, R. Ion Hydration and Association in Aqueous Potassium Phosphate Solutions. *J. Phys. Chem. B* **2015**, *119*, 5270–5281.

(S2) Marcus, Y. *Ion Properties*; CRC Press: Boca Raton, USA, 1997.

(S3) Hubbard, J. B.; Colonomos, P.; Wolynes, P. G. Molecular Theory of Solvated Ion Dynamics. III. The Kinetic Dielectric Decrement. *J. Chem. Phys.* **1979**, *71*, 2652–2661.

(S4) Sega, M.; Kantorovich, S.; Arnold, A. Kinetic Dielectric Decrement Revisited: Phenomenology of Finite Ion Concentrations. *Phys. Chem. Chem. Phys.* **2015**, *17*, 130–133.
